# Minimally Invasive Removal of Frontal Lipoma with Endoscope

**DOI:** 10.1007/s00266-025-05293-x

**Published:** 2025-10-10

**Authors:** Yimou Sun, Qifei Wang, Hongbin Xie

**Affiliations:** Department of Plastic Surgery, Peking University Third Hospital, Haidian District, 49 North Garden Road, Beijing, People’s Republic of China

**Keywords:** Forehead, Lipoma, Endoscope, Scar, Minimal invasion

## Abstract

**Background:**

Frontal lipomas are commonly excised through an overlying incision. Endoscopic techniques can be used to remove the lesions for a cosmetic outcome.

**Objectives:**

This study aimed to evaluate the outcomes of endoscopic removal of frontal lipomas and summarize the associated experiences.

**Methods:**

Eighty-three patients with forehead lipomas were included in the retrospective study. Fifty-six patients underwent endoscopic surgery (endoscopic excision group), while twenty-seven patients underwent direct excision (direct excision group). Patients characteristics including age, gender, tumor locations and sizes were collected. Additionally, surgery duration, surgery costs, hospital costs, satisfaction with the procedure, complications and follow-up were also evaluated.

**Results:**

Patients in endoscopic excision group were younger than those in direct excision group. Additionally, the average surgery duration in endoscopic excision group was longer compared to that in direct excision group. Patients in endoscopic excision group were more satisfied with smoothness of appearance and were more than willing to recommend endoscopic surgery to others. There was no significant difference in satisfaction regarding pain or discomfort control and hospitalization costs between the two groups. No complications such as wound infection, poor wound healing, forehead scars and recurrence were observed in endoscopic excision group. No noticeable scars were left on the forehead in the endoscopic excision group compared to the direct excision group.

**Conclusions:**

Endoscopic excision is a safe and minimally invasive approach for forehead lipomas.

**Level of Evidence II:**

This journal requires that authors assign a level of evidence to each article. For a full description of these Evidence-Based Medicine ratings, please refer to the Table of Contents or the online Instructions to Authors  www.springer.com/00266.

**Supplementary Information:**

The online version contains supplementary material available at 10.1007/s00266-025-05293-x.

## Introduction

Lipomas are common benign subcutaneous lesions, with an incidence rate of approximately 0.1%. They can develop in various parts of the body, with the trunk and limbs being particularly frequent locations. Frontal lipomas account for about 13.3% of cases [1]. Typically, lipomas grow within the superficial subcutaneous fat layer. However, frontal lipomas may be located in the loose connective tissue between the frontal muscle and the periosteum [2] or may occur within the frontal muscle itself or between the frontal muscle and its deep fascia [3]. Ultrasonography has been shown to have an accuracy rate of less than 70% in distinguishing whether a lipoma is superficial or deep [4]. In certain cases, further imaging confirmation using CT or MRI is necessary. Although most lipomas are benign, lipomas in deeper locations may undergo malignant transformation into liposarcomas. Additionally, compressive symptoms may occur, when lipomas grow too large and press on nearby tissues. Moreover, lipomas on the face can affect one's appearance and also cause psychological stress. Hence, lipomas especially located in forehead arouse everyone's concern.

The classical surgical approach for excising frontal lipomas typically involves direct excision [5, 6]. However, this method carries the risk of scarring in the frontal area, which can be particularly troublesome and frustrating for patients with large masses, low-lying positions, smooth and wrinkle-free skin, or a predisposition to scarring. Additionally, a transverse incision in the frontal region may damage the vessels and nerves that run vertically, leading to complications such as bleeding, hematoma and numbness in the surgical site and scalp [7]. In recent years, endoscopic-assisted surgery has gained popularity due to its minimally invasive nature. This approach is associated with fewer complications, reduced postoperative pain and bleeding, earlier mobilization and shorter hospital stays [8]. A significant advantage of endoscopic techniques is that incisions are often made in concealed locations, effectively addressing the concern of visible surgical scars [9].

Endoscopy has been effectively utilized in plastic surgery, particularly in procedures such as breast augmentation [10], facial wrinkle reduction [11] and brow lifts [12], all yielding safe and satisfactory outcomes. While a few articles have documented cases of endoscopic-assisted excision of frontal lipomas, they often lack comprehensive surgical details [13, 14].

In this study, experiences and insights from 56 cases of endoscopic frontal lipoma surgery are summarized. A standardized surgical procedure and an optimal approach are proposed. Additionally, treatment satisfaction and complications compared to direct excision surgery were also investigated. It is expected that the technique will significantly improve surgical efficiency, reduce complications, minimize forehead scarring, enhance aesthetic outcomes and ultimately increase patient satisfaction.

## Patients and Methods

### Patients

**Fig. 1:**
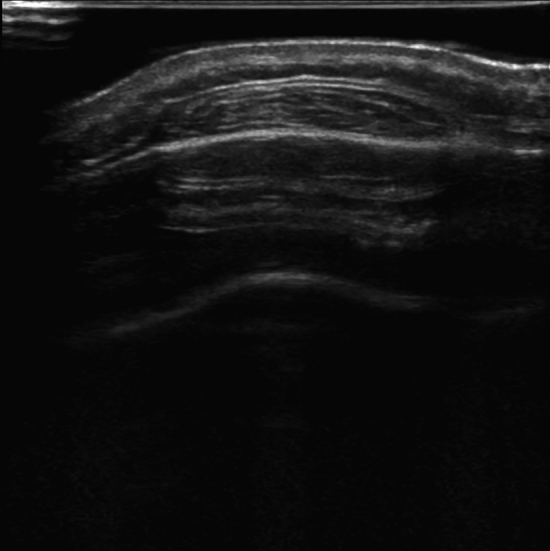
Ultrasound results of forehead lipoma

Eighty-three patients with forehead lipomas from January 2019 to January 2023 were included in the retrospective study. Fifty-six patients (35 females and 21 males) underwent endoscopic surgery (endoscopic excision group), while 27 patients (19 females and 8 males) underwent direct excision (endoscopic excision group). Preoperative ultrasound examinations were conducted (Fig. 1), and the diagnoses were jointly confirmed by ultrasound and plastic surgeons. All excised tumor specimens were histologically analyzed for pathological diagnosis. Additionally, the study has been approved by the appropriate institutional and national research ethics committee and has been performed in accordance with the ethical standards as laid down in the 1964 Declaration of Helsinki and its later amendments or comparable ethical standards.

Inclusion criteria: Adults (≥16 years), maximal diameter measured 0.5–5 cm via preoperative ultrasonography, ASA physical status I-II, provide informed consent. Exclusion criteria comprised: lesions suspicious for malignancy; lipoma sizes unsuitable for endoscopic resection (e.g., size <0.5 cm or >5 cm, high-risk locations); significant comorbidities (ASA ≥III, uncontrolled coagulopathy); inability to discontinue anticoagulants; pregnancy/lactation; or syndromic lipomatosis.

### Anatomy

**Fig. 2:**
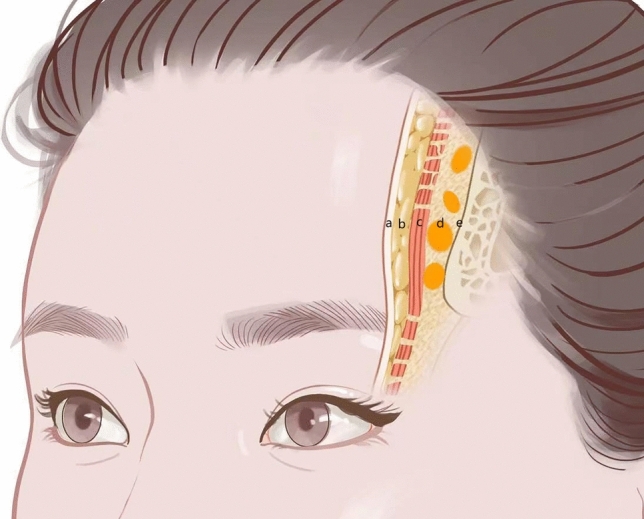
Schematic diagram of frontal anatomy. Forehead is classified into five layers: skin **a**, subcutaneous tissue **b**, frontalis muscle **c**, loose connective tissue beneath the frontalis muscle **d** and periosteum **e**

The forehead is anatomically regarded as an extension of the scalp. Traditionally, it has been classified into five layers: skin, subcutaneous tissue, frontalis muscle (an extension of the aponeurotic fascia), loose connective tissue beneath the frontalis muscle and periosteum (Fig. 2). Recent research, however, has proposed a more detailed classification, dividing the forehead into eight distinct layers: skin, subcutaneous tissue, superficial fascia of the frontalis muscle, frontalis muscle-aponeurotic fascia, deep fat compartment beneath the frontalis muscle, deep fascia of the frontalis muscle, loose connective tissue beneath the frontalis muscle and periosteum [15]. The subcutaneous tissue in the frontal area is thin and dense, lacking apparent gaps. The frontalis muscle is composed of two thin muscular sheets that originate from the galea aponeurotica and merge with the corrugator supercilii muscle and the orbicularis oculi muscle in an oblique inner-to-lower outer direction. The existence of deep fat compartments has also been confirmed through contrast-enhanced computed tomographic scans and anatomical dissections, with these deep forehead compartments generally being avascular planes [16].

The loose connective tissue layer between the frontalis muscle and the periosteum is recognized for providing an easily dissectible space, which allows for movements such as brow elevation and frowning. Blood supply to the medial orbital and central frontal areas is provided by the supraorbital artery and supratrochlear artery, which emerge at the supraorbital notch and supratrochlear incision, respectively, and course upward from deep to superficial. The lateral frontal area is supplied by the superficial temporal artery. It has been observed that the blood supply to the frontal area is abundant, with numerous anastomoses and variations present among different arteries. The primary nerve supply to the frontal area is derived from the ophthalmic division of the trigeminal nerve, including the supratrochlear nerve and the supraorbital nerve. The supratrochlear nerve is primarily responsible for providing sensory innervation to the central frontal area, while the superficial branch of the supraorbital nerve supplies sensory innervation to the broader frontal region. Additionally, the deep branch of the supraorbital nerve runs cephalically in the subfascial plane of the lateral frontal area, supplying sensory innervation to the skin at the vertex.

### Equipment

**Fig. 3:**
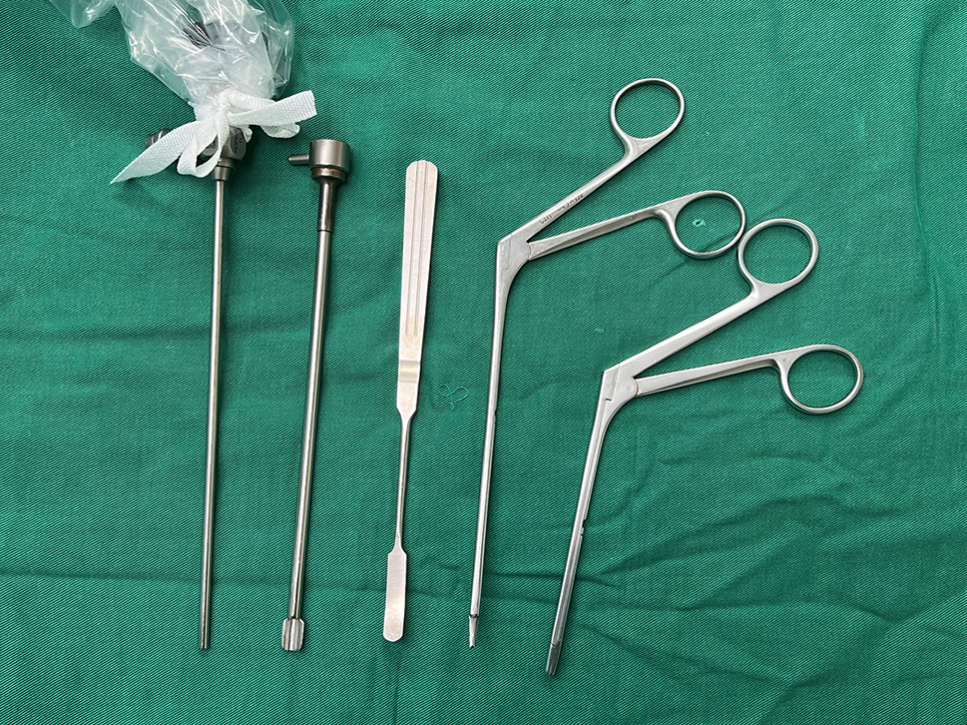
Endoscopic surgical instruments. These instruments (from left to right) include endoscope, endoscope sheath, nasal speculum, endoscopic scissors and endoscopic forceps

The endoscopic forehead lipoma resection procedure requires specialized instruments that are not typically used in open surgical procedures. The endoscopic procedures were performed using a dedicated STORZ endoscopic imaging system (IMAGE1 S™, Karl Storz GmbH, Tuttlingen, Germany) coupled with a 4-mm-diameter 30° Hopkins II telescopic endoscope (Model 26003BA). The system provided HD 1080p resolution for optimal visualization. A 4-mm disposable valved trocar sheath (Applied Medical, Rancho Santa Margarita, CA) was used for instrument access. Subperiosteal dissection was achieved using four graded periosteal elevators (0°, 10°, 20°, 30° curvature; Martin GmbH, Tuttlingen, Germany) with 2.7 mm working tips to accommodate the 4 mm working channel. Tissue manipulation employed 5-Fr endoscopic bipolar forceps (with rotational capability) and 3 mm curved endoscopic scissors (both from STORZ). All instruments were sterilized using hydrogen peroxide plasma (STERRAD® NX) between procedures (Fig. 3).

### Endoscopic Surgical Method

**Fig. 4:**
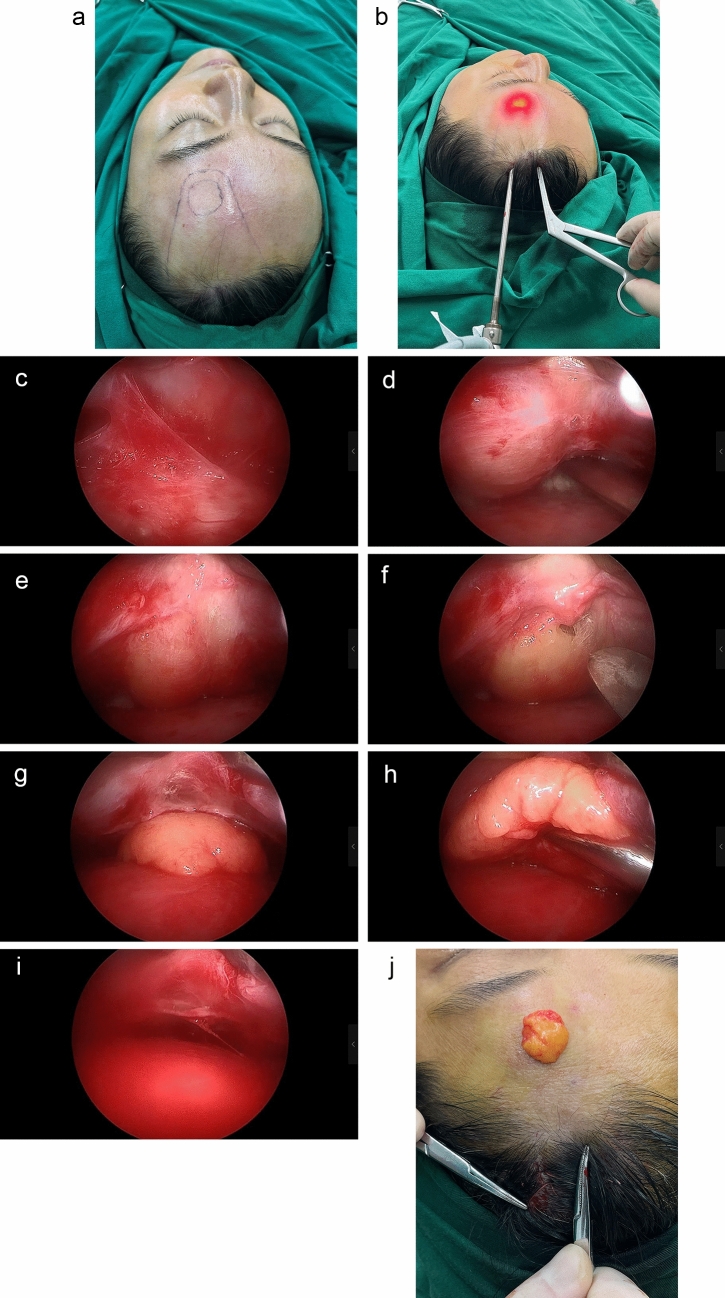
General steps of endoscopic surgery. Preoperative marking and anesthesia coverage **a**, endoscopic surgery external view **b**, surgical procedure in endoscopic imaging system **c–i** and tumor resection completion **j**. The sizes in image 4a and 4j are 2.1 × 2.3 cm (preoperative measurement) and 2.6 × 2.9 cm (intraoperative measurement), respectively

In patients treated with endoscope, 5 cases were conducted under intravenous anesthesia, while the remaining 51 cases were performed under local anesthesia. Two 1 cm incisions were made within 0.5 cm of the hairline. These incisions were positioned 5 cm from the midline and parallel to the axis of the hair follicles to avoid any negative impact on hair growth. All patients received local infiltration anesthesia with a solution of 1% lidocaine and 1:400,000 epinephrine. The infiltration area extended 0.5 cm beyond the two incisions and included the connecting area to the lipoma, forming a triangular region known as the dissection triangle. Anesthesia was targeted at the loose connective tissue layer beneath the frontalis muscle, facilitating dissection and minimizing bleeding. Endoscopic dissection was performed along this layer using a periosteal elevator, extending from the two incisions to the location of the lipoma, thereby completely separating the dissection triangle. Unnecessary dissection in the subperiosteal layer was avoided to minimize surgical steps, reduce procedural complexity and enhance efficiency. Upon reaching the location of the lipoma, the frontalis-associated lipoma was visualized under the endoscope, covered by fascia. The fascia enveloping the lipoma was meticulously separated using endoscopic forceps or an elevator to fully liberate the lipoma. The lipoma was then grasped with endoscopic forceps and extracted through the incisions. For superficial subcutaneous lipomas, the frontalis muscle was separated using endoscopic forceps to expose the lipoma prior to dissection and extraction. The surgical site was irrigated and examined to confirm the absence of residual tissues or bleeding. The aponeurotic fascia was closed with 4-0 absorbable suture material to minimize tension, and the incision was closed with interrupted sutures using 5-0 monocryl. The placement of a drain was deemed non-mandatory and depended on the surgeon's preference; the author routinely opted not to use drainage tubes. An adequate pressure dressing was applied to the forehead postoperatively to mitigate bruising and swelling within 24 hours after surgery (Fig. 4 and Video 1).

### Postoperative Care

Antibiotics were not required after the surgery. The dressing was removed 24 hours after surgery, the wound was disinfected, and scabs were removed. If a drain was inserted, it was also removed on this day. Mild swelling of the forehead was expected in the early postoperative period, but it usually resolved within 3–5 days. Some patients reported pain in the surgical area, which could be alleviated with oral analgesics. Suture removal was performed on day 7 after the surgery. Follow-up appointments were scheduled at 1 month, 3 months, 6 months, 1 year and 3 years postoperatively.

### Statistical Analysis

SPSS 24.0 statistical software was used for data analyses. Continuous variables were expressed as mean ± standard error. Categorical variables were summarized with number and percentage of patients. The t test was performed to analyze differences between two groups. *P*<0.05 was considered to be statistically significant.

## Results

### Patient Characteristics

**Fig. 5:**
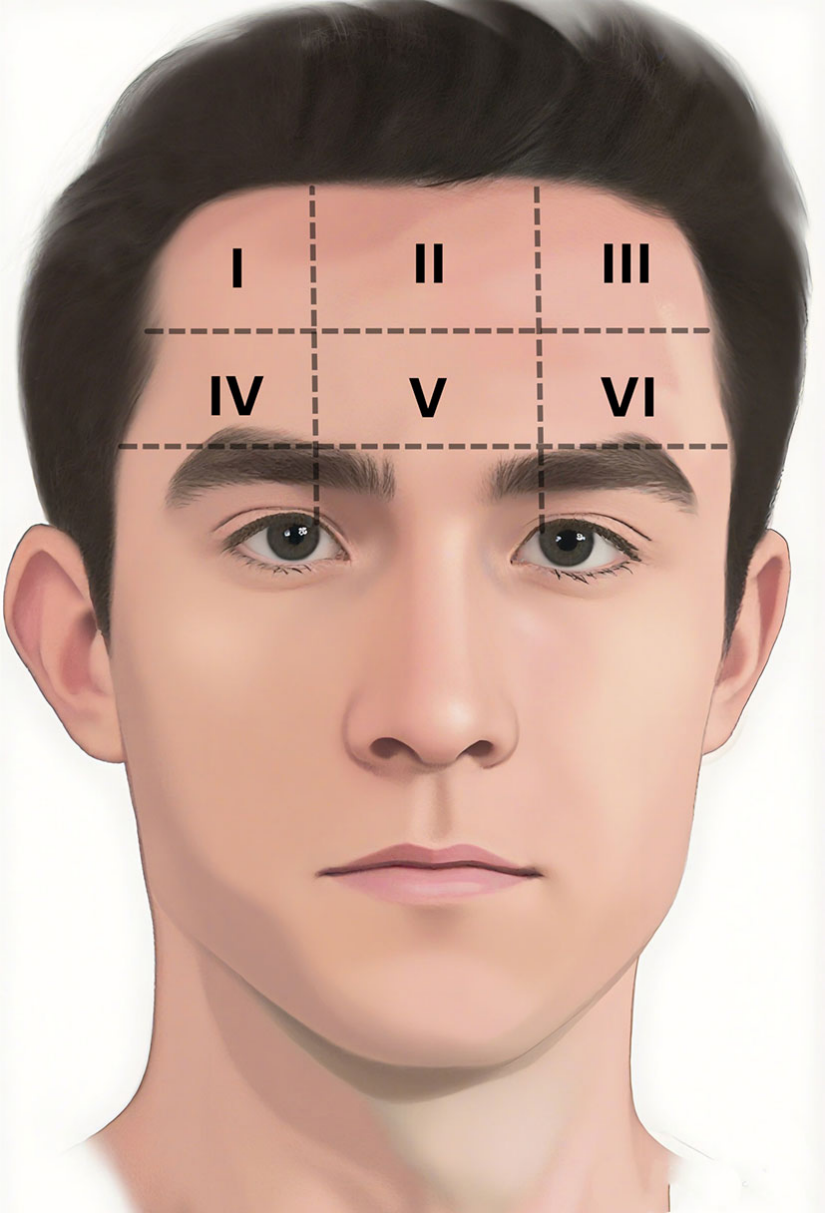
A schematic diagram illustrating the distribution of lipomas. The forehead is systematically divided into six zones (I-VI) by three horizontal lines (hairline, bilateral supraorbital ridge line 2 cm above the brow and inter-brow ridge line) and two vertical lines along the medial iris margins. The number of lipomas in the six zones among 83 patients was 18 (21.7%), 14 (16.9%), 17 (20.5%), 6 (7.2%), 19 (22.9%) and 9 (10.8%), respectively

Eighty-three patients suffering with forehead lipomas were included in the study. Fifty-six patients (35 women and 21 men) underwent endoscopic surgery. Twenty-seven patients (19 women and 8 men) underwent direct excision surgery. A schematic diagram illustrating the distribution of lipomas among all patients is provided in Fig. 5. Their basic information, including gender, age, tumor location, tumor size and surgery duration, is documented in Table 1. There is no difference in gender and tumor location between endoscopic excision group and direct excision group (*P*>0.05). Patients in endoscopic excision group were younger than those in direct excision group (*P*=0.027). Additionally, the average surgery duration in endoscopic excision group was longer compared to that in direct excision group (*P *< 0.001).

**Table 1:** Patients basic information

Variable	Endoscopic excision group	Direct excision group	*P*
Gender			
Female	35	19	0.481
Male	21	8	
Age (years)	34.4±9.2	38.6±8.9	0.027
Tumor location			
Left forehead	18	9	0.08
Middle forehead	11	5	
Right forehead	27	13	
Tumor size (cm)	2.3×1.6×1.3	1.7×1.9×1.4	/
Surgery duration (minutes)	44.6±11.5	32.7±7.2	*P*<0.001

### Surgery Satisfaction Questionnaire

The surgery satisfaction questionnaire is summarized in Table 2. The questionnaire primarily includes pain or discomfort control, smoothness of appearance, forehead scar prevention, surgery costs and hospitalization costs. Patients in endoscopic excision group were more satisfied with smoothness of appearance because of scar-free forehead. They are more than willing to recommend endoscopic surgery to others. Pain control satisfaction in two group is completely consistent. Additionally, there was no significant difference in satisfaction with hospitalization costs in the two groups.

**Table 2:** The surgery satisfaction questionnaire

Variable	Endoscopic excision group			Direct excision group	
	No of patients	No.(%)		No. of *p*	No.(%)
Are you satisfied with pain or discomfort control?	56			27	
Not at all		0(0)			0(0)
Somewhat		0(0)			0(0)
Moderately		0(0)			0(0)
Very much		0(0)			0(0)
Extremely		56(100)			27(100)
Are you satisfied with smoothness of appearance?	56			27	
Not at all		0(0)			0(0)
Somewhat		0(0)			1(3.7)
Moderately		0(0)			2(7.41)
Very much		0(0)			4(14.81)
Extremely		56(100)			20(74.07)
Are you satisfied with hospitalization costs*?	56			27	
Not at all		0(0)			0(0)
Somewhat		0(0)			0(0)
Moderately		0(0)			0(0)
Very much		2(3.57)			1(3.7)
Extremely		54(96.43)			26(96.3)
Are you satisfied with surgery costs**?	56			27	
Not at all		0(0)			0(0)
Somewhat		2(3.57)			0(0)
Moderately		4(7.14)			3(11.11)
Very much		6(10.71)			4(14.81)
Extremely		44(78.57)			20(74.07)
Would you recommend this surgical procedure to others?	56			27	
Not at all		0(0)			0(0)
Somewhat		0(0)			0(0)
Moderately		0(0)			2(7.41)
Very much		1(1.79)			4(14.81)
Extremely		55(98.21)			21(77.78)

^*^Hospitalization fees refer to the costs incurred while a patient is admitted to the hospital. This includes charges for room and board, nursing care, daily tests, medications and meals

^**^Surgical fees are specifically related to the costs associated with a surgical procedure. This includes charges for the surgery itself, anesthesia, the use of surgical instruments and the operating room

### Surgical Outcome and Complications

**Fig. 6:**
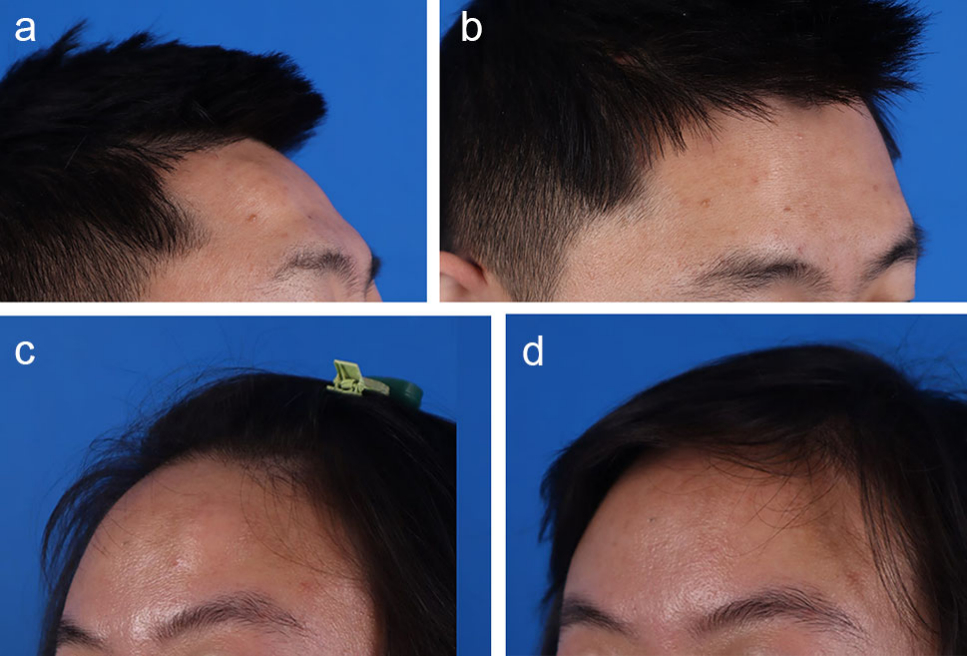
Aesthetic outcomes of endoscopic approach for forehead lipoma excision. Preoperative images **a,c** and postoperative images **b,d**

Frontal lipomas in endoscopic excision group were successfully removed via endoscope without the need to convert to the conventional open surgery. The soft tissue tumors were completely removed with their capsular walls intact. No complications such as wound infection, poor wound healing, scar, alopecia, numbness, hematoma, swelling, fat liquefaction and recurrence were observed in endoscopic excision group (Table 3). While tumors in direct excision group were excised through a direct skin incision, noticeable scars were left on the forehead (Fig. 6), which is significantly different from the results observed in the endoscopic group (*P *< 0.001).

**Table 3:** Surgical complications

Complications	Endoscopic excision group	Direct excision group	*P*
Wound infection	0	0	1
Poor wound healing	0	0	1
Visible scars	0	19	P<0.001
Alopecia	0	0	1
Numbness	0	0	1
Hematoma	0	0	1
Swelling	2	1	0.826
Fat liquefaction	0	0	1
Recurrence	0	2	1
Total	2	22	*P *< 0.001

Logistic regression was performed to adjust for confounding factor age

## Discussion

Forehead lipomas, classified as benign tumors, represent a particularly troublesome location for patients. The forehead is a prominent area of the face, and traditional surgical excision may be rejected by patients due to the resultant scars. The endoscopic technique effectively addresses the issue of visible scars on the forehead by concealing the incisions within the hairline. Furthermore, the utilization of an endoscope for deep dissection and manipulation beneath the frontal muscle protects the blood vessels and nerves that are longitudinally distributed in the forehead area, thereby reducing the likelihood of postoperative bleeding and numbness. This approach achieves minimally invasive, scarless procedures with rapid recovery and few complications.

The design of the incision takes multiple factors into account. Primarily, it should be concealed within the hairline. For male patients who may experience hair loss, the incision may be positioned further from the hairline. Although some literature suggests the use of a single incision for the procedure [14, 17], it is believed that two incisions represent the optimal choice. The two incisions, combined with the lesion’s location, form an isosceles triangle with a base measuring 4–5 centimeters. One incision is utilized for the insertion of the endoscope with a sheath, while the other incision is designated for instrument placement. This design facilitates the flexible and convenient execution of all surgical procedures.

Opinions vary regarding the level of separation within the visual space. Some surgeons opt for subperiosteal separation [18]. However, separation above the periosteum is strongly recommended. The anatomical layer of frontalis-associated lipoma is located beneath the frontal muscle and above the periosteum, making this the most accessible layer for direct separation. This approach ensures the integrity of the periosteum while enhancing surgical efficiency. All surgeries were performed at this separation layer.

Postoperative complications are exceedingly rare with this technique. In a three-year follow-up of 56 patients, no cases of poor wound healing, numbness or recurrence were observed. The application of endoscopic techniques enhances the minimally invasive nature of the procedure and promotes postoperative recovery. Proficient knowledge of anatomy and exceptional surgical skills are essential prerequisites for minimizing complications. Complete removal of the lipoma is crucial in reducing the likelihood of recurrence. Operating at the supraperiosteal level effectively avoids injury to superficial blood vessels and nerves.

There are a few points regarding our research that need to be emphasized. After understanding the advantages and disadvantages of direct excision and endoscopic surgery to the participants, the young individuals chose endoscopic surgery. They are more concerned about their appearance, significantly reducing scarring, and are less focused on the cost of the surgery, even though the cost of endoscopic surgery is much higher than that of direct excision. Lipomas can occur at any age, but are more commonly seen in individuals aged 40–60 years. They can occur in areas such as the head, neck, back and proximal limbs. They are more common in males [19]. However, contrary to previous reports, the incidence of lipomas in our study is higher in females than in males. The reason may be due to the different locations of occurrence in the surveyed population; our study focused solely on the incidence in the frontal area, while other studies included various locations throughout the body. Additionally, the willingness to seek medical attention may vary; lipomas located on the forehead can affect appearance, leading female patients to be more proactive in seeking treatment.

Endoscopic patients were younger (34.4 ± 9.2 vs 38.6 ± 8.9 years; *P*=0.027). Age may confound scar/satisfaction outcomes. To rigorously address this concern, we performed ANCOVA adjusting for age to evaluate its influence on the primary outcomes. After age adjustment, the between-group differences remained statistically significant (P < 0.05), indicating that age did **not** substantially confound the outcomes. We explicitly discuss age as a potential confounder. While younger patients predominated in the endoscopic cohort, our adjusted analysis suggests that the superior scar/satisfaction outcomes are likely attributable to the surgical technique itself rather than age disparities. Biological plausibility is acknowledged but deemed unlikely to alter our conclusions, given the statistical findings.

One limitation of this procedure is its dependence on endoscopic equipment. Proficiency in endoscopic techniques must be acquired by surgeons through dedicated learning and training, which establishes a certain technical threshold. Some patients may be unfamiliar with this surgical approach or may harbor doubts regarding the complete removal of the lipoma, necessitating effective patient education. Furthermore, endoscopic surgery is typically more expensive than traditional open surgery, making it more suitable for patients with greater economic ability.

## Conclusion

Endoscopic forehead lipoma resection is a technique worthy of promotion. The technique significantly enhances surgical efficiency, minimizes the risk of forehead scarring, improves aesthetic outcomes and increases patient satisfaction.

## Supplementary Information

Below is the link to the electronic supplementary material.

Video of forehead lipoma excision via endoscope (MP4 90724 kb)
